# Barriers and facilitators to medicine collection through the CCMDD programme at a Durban Hospital

**DOI:** 10.4102/hsag.v27i0.1906

**Published:** 2022-09-27

**Authors:** Simangele I. Hlongwana, Andrew L. Gray

**Affiliations:** 1 Discipline of Public Health Medicine, University of KwaZulu-Natal, Durban, South Africa; 2 Discipline of Pharmaceutical Sciences, School of Health Sciences, University of KwaZulu-Natal, Durban, South Africa; 3 King Edward VIII Hospital, Durban, South Africa; 4 Division of Pharmacology, Discipline of Pharmaceutical Sciences, University of KwaZulu-Natal, Durban, South Africa

**Keywords:** CCMDD, barriers and facilitators, chronic medicine collection, patient medicine parcels, pick-up-points, National Health Insurance, patient characteristics, adherence

## Abstract

**Background:**

South Africa has rekindled health reform efforts through the implementation of the Centralised Chronic Medicine Dispensing and Distribution (CCMDD) programme, as a precursor towards achieving envisioned National Health Insurance (NHI). The CCMDD programme enables stable patients to collect chronic medicines dispensed centrally from designated pick-up-points (PuPs). Barriers and facilitators of chronic medicine collection exist at different levels.

**Aim:**

To identify barriers and facilitators associated with patients’ characteristics and noncollection of CCMDD patient medicine parcels (PMPs).

**Setting:**

The study was conducted at a regional public sector hospital which provides support for 19 primary facilities.

**Methods:**

An observational cross-sectional comparative study was conducted.

**Results:**

There was no statistically significant difference in collection status in terms of most of the variables compared. Patients who had been on treatment longer or who were receiving multiple items were more likely to collect medication, as were patients with arthritis, HIV and AIDS, but the association was no longer significant after adjusting for other confounders. Patients using internal PuPs were significantly more likely to collect their PMPs than patients using external PuPs, and this may have implications for achieving CCMDD objectives.

**Conclusion:**

This study has revealed that recently diagnosed patients are enrolled onto the CCMDD programme whilst the chronic condition stability is not yet attained. Patients were also enrolled onto the programme at the referral facility instead of being down-referred.

**Contribution:**

This study makes a case for evaluation research to further assess the CCMDD programme implementation, in order to improve uptake and cost-effectiveness.

## Introduction

The noticeable rise in noncommunicable disease (NCD) prevalence in South Africa has necessitated the implementation of health reforms, with a shift towards comprehensive chronic disease management (Meyer 2017). The 2017 Global Burden of Disease (GBD) study revealed that globally, NCDs accounted for 73.4% of total deaths, whilst communicable, maternal, neonatal and nutritional conditions accounted for 18.6% and injuries 8.0% (Harikrishnan et al. 2018). The 2017 GBD study also found that the overall number of deaths due to NCDs increased globally between 2007 and 2017, whilst the mortality rate decreased from 582.1 deaths per 100 000 to 536.1 deaths per 100 000. In high-income countries (HICs), innovative strategies to improve access to chronic medicine have included appointment-based medication synchronisation, less frequent clinic appointments, delivery of chronic medicine to patients’ homes, adjusted pharmacy operating hours and phone-in, mail order or Internet prescription refill (Hersberger & Messerli 2016; Holdford & Saxena 2015; Holtzman et al. 2015; Rolnick et al. 2013). In low- and middle-income countries (LMICs), adherence clubs have been used as an alternative strategy to enable patients to collect chronic medicine (Venables et al. 2016). Models similar to those implemented in HICs, such as less frequent clinic appointments and home deliveries, have also been implemented in sub-Saharan African countries (Geldsetzer, Ortblad & Bärnighausen 2016).

The main objective of implementing decentralised chronic patient management strategies in LMICs was to improve efficiency of the health system (Bemelmans et al. 2014). This has proven beneficial for improving patients’ adherence to treatment, reducing transport costs and minimising time away from work, as the services are brought closer to patients’ homes (Bemelmans et al. 2014; Geldsetzer et al. 2016; Venables et al. 2016). The implementation of these models was initially focused on stable patients on antiretroviral therapy (ART), but due to the increased number of patients with comorbidities, integrated models of chronic care have become necessary (Magadzire, Marchal & Ward 2016). These initiatives have involved the predispensing of repeat chronic prescriptions with hospital-level medicine as per standard treatment guidelines and essential medicine list (STG and EML) at hospital for issuing at the primary health care (PHC) and/or at community level. Chronic disease management was further strengthened by enhancing the capacity of PHC nurses in managing patients living with chronic conditions and increasing the basket of chronic medicine that can be accessed at the primary level.

In line with the World Health Organization’s (WHO) differentiated care model (World Health Organization 2016), the South African public sector has piloted the integrated chronic disease management (ICDM) model at PHC level, incorporating it into the Ideal Clinic initiative in 2014 (Mwagomba et al. 2018). A number of initiatives have been introduced in preparation for National Health Insurance (NHI), including the Centralised Chronic Medicine Dispensing and Distribution (CCMDD) programme. The CCMDD programme was implemented nationally in February 2014, as part of the ICDM Framework (Dlamini 2018; Magadzire, Marchal & Ward 2015; Meyer 2017). Initially, bids to provide a centralised dispensing and distribution service for chronic medicines in 10 NHI pilot districts in 8 of the 9 provinces were invited (Bradley et al. 2016; Du Toit 2014). In 2016, the CCMDD programme was expanded to 46 districts across the 8 participating provinces (Meyer 2017). Centralised or distance dispensing services, by courier pharmacies, have been used widely in the South African private sector (Magadzire et al. 2015).

The CCMDD programme enables stable patients to collect chronic medicines from designated internal or external pick-up-points (PuPs) (Du Toit 2017). Internal PuPs refer to public sector health facilities, whilst external PuPs refer to private sector community pharmacies or general practitioners, community-operated adherence clubs or community-based service points, such as church halls. Missed appointments are a global challenge for all chronic disease management processes and have a negative impact on patient clinical health outcomes, resulting in a potential increase in the cost of health service delivery (Magadzire, Mathole & Ward 2017). Between 8% and 12% of patient medication parcels (PMPs) distributed by the Western Cape Central Dispensing Unit (WC CDU) were not collected, and only about 43% of patients on ART enrolled in the CCMDD programme in the Eastern Cape collected their PMPs (Katende-Kyenda 2018; Magadzire et al. 2017). The PuPs are expected to notify the CCMDD service provider when a patient fails to collect within 48 h of a scheduled collection date, in order to trigger the tracing process. Uncollected PMPs result in avoidable financial costs, medicine expiration and administrative burdens (Magadzire et al. 2015).

Pharmacy refill and claims data are amongst the indirect measures of patients’ adherence to treatment. There is no universally agreed-upon best method of assessing adherence, but there is an assumption when using a refill gap algorithm that patients are taking the medication as prescribed (Amir et al. 2018; Pinto et al. 2018). This study indirectly measured adherence by assessing patient chronic medicine collection behaviours. Although some studies on patient adherence to chronic medicines have been conducted in South Africa, most have focused on ART, rather than NCDs (Katende-Kyenda 2018; Magadzire et al. 2017; Rubio-Valera et al. 2014). None have yet been conducted in the KwaZulu-Natal (KZN) context. Such studies are critical for designing interventions that are context-specific, covering barriers and facilitators at all levels: interpersonal, intrapersonal, institutional, community and public policy levels (Rubio-Valera et al. 2014). The aim of this study was to assess barriers and facilitators to medicine collection through the CCMDD programme at a regional facility in KZN in 2017. The specific objectives were to describe characteristics of patients registered on the programme, compare and assess any association between patient’s characteristics and noncollection of CCMDD PMPs.

## Research methods and design

### Design and setting

An observational cross-sectional comparative study was conducted at a regional public sector hospital located in Umlazi community area. A purposive, nonprobability convenience sampling approach was followed; the facility was in close proximity to the researcher’s work area. The hospital provides support for 19 PHC facilities. The hospital implemented the CCMDD programme in February 2016. Patient medication parcel collection streams were provided via a facility fast lane (internal) and outsourced external PuPs, including adherence clubs.

### Study population and sampling strategy

Data obtained from the CCMDD service provider database revealed that between April and September 2017, a total of 31 157 patients received chronic medicines through the CCMDD programme managed by this hospital. Of these, 30 382 patients were recorded on the centralised dispensing service provider database as having collected their PMPs (referred to as ‘collectors’), whilst 777 were recorded as ‘noncollectors’. All patients who were registered to the programme during the study period, as per the service provider database, were included in the study population. Patients whose files and/or CCMDD prescription forms were missing after being sampled from the service provider database were excluded. A sample size of 160 patient files (80 collectors and 80 noncollectors) was determined by the statistician, based on an estimated difference of 20% (equivalent to an odds ratio [OR] of 2.5) between the two groups, a power of 85% and a probability of 95%. A total of 164 patient files were systematically sampled (*n* = 80 ‘collectors’ and *n* = 84 ‘noncollectors’). The total CCMDD population per month was used to determine the sample size proportionate to the patient volume, and the proportionate number per month was selected until the sample size per group was reached.

### Data collection

Data were extracted from the CCMDD service provider database and the patient medical records by the lead researcher through retrospective medical record reviews. This was also done to improve reliability. The variables collected were as per attached (Table 1-A1). The data were captured on Excel during data collection by the researcher, who is competent in Excel, and analysed by the qualified statistician.

### Data analysis

Frequency distributions of continuous data were examined for normality using Shapiro–Wilk tests and means (standard deviation [SD]) and medians (interquartile ranges [IQR]) used as appropriate. Comparisons were made using the chi-squared test, Student’s *t*-test or Wilcoxon rank-sum test. Factors associated at the bivariate level were included in a logistic model to determine independent risk factors. Logistic regression analysis was used to analyse the characteristics of collectors compared to those of noncollectors to control for confounders. Data were analysed using Stata version 13.1.

### Ethical considerations

Ethical approval was obtained from the University of KwaZulu-Natal (UKZN) Biomedical Research Ethics Committee (reference number: BE558/18). No patient identifiers were recorded in order to maintain patient confidentiality.

## Results

### Demographic characteristics

The mean age of the sample of 164 patients registered on the CCMDD programme in this hospital was 55.0 years (varied from 13–95 years, SD 18.7), with female patients constituting the majority (60.4%; 99/164) (Table 1a). There was no statistically significant difference in collection status between age groups (*p* = 0.8). Female patients (50.5%; 50/99) were slightly more likely to collect PMPs dispensed through the CCMDD programme than their male (46.2%; 30/65) counterparts, but the difference was not statistically significant (*p* = 0.6).

**TABLE 1a:** Patients’ demographics and clinical characteristics by collection status.

Demographics and clinical characteristics	Total (*n* = 164)		Collected (*n* = 80)		Not collected (*n* = 84)	
	*n*	%	*n*	%	*n*	%
**Age group** †						
0–14	4	2.4	3	75.0	1	25.0
15–24	7	4.3	4	57.1	3	42.9
25–54	67	40.9	30	44.8	37	55.2
55–64	30	18.3	15	50.0	15	50.0
> 65	56	34.1	28	50.0	28	50.0
**Sex**						
Female	99	60.4	50	50.5	49	49.5
Male	65	39.6	30	46.2	35	53.8
**Place of residence**						
Outside community area	57	34.8	25	43.9	32	56.1
Within community area	107	65.2	55	51.4	52	48.6
**Occupation**						
Employed	31	18.9	14	45.2	17	54.8
Pensioner	72	43.9	38	52.8	34	47.2
Below employable age*	11	6.7	8	72.7	3	27.3
Unemployed	28	17.1	12	42.9	16	57.1
Not indicated	22	13.4	8	36.4	14	63.6
**Comorbidity**						
Yes	100	61.0	51	51.0	49	49.0
No	63	38.4	29	46.0	34	54.0
No record	1	0.6	0	0.0	1	100.0
**Clinical outcome**						
Negative	45	27.4	22	48.9	23	51.1
Positive	116	70.7	57	49.1	59	50.9
No record	3	1.8	1	33.3	2	66.7

SD, standard deviation.

† , Mean 55.0061 (SD 18.73401).

* , *p* ≤ 0.05.

Most (65.2%; 107/164) patients resided within the community area, with pensioners, below employable age and employed constituting 43.9% (72/164), 6.7% (11/164) and 18.9% (31/164), respectively. The patients living within the community area were more likely to collect (51.4%; 55/107) compared to their counterparts from outside the community area (43.9%; 25/57), but this difference was also not statistically significant (*p* = 0.85). Those below employable age were most likely to collect (72.7%; 8/11) their CCMDD PMPs, followed by pensioners (52.8%; 38/72), whilst the majority of unemployed patients (57.1%; 16/28) did not collect their CCMDD PMPs. Nonetheless, there was no statistically significant difference in the proportion of collectors and noncollectors by employment status (*p* = 0.3) (Table 1a).

### Clinical characteristics

Patients with comorbid conditions (51.0%; 51/100) were slightly more likely to collect compared to those without comorbidity (46.0%; 29/63), but the difference was not statistically significant (*p* = 0.5). A positive clinical outcome was recorded in 116/164 (70.7%) patients. In the context of this study, negative clinical outcomes included uncontrolled hypertension, uncontrolled diabetes, a record of acute asthma attacks or episodes of seizures in patients treated for epilepsy. Those who were recorded as having negative clinical outcomes were less likely to collect (51.1%; 23/45) compared to those who had positive clinical outcomes (50.9%; 59/116) (*p* = 0.9), but the difference was not statistically significant.

### Prescription records and disease conditions

A total of 192 prescriptions were recorded for the sample of 164 patients. However, as each prescription could record up to three different disease conditions, 268 disease conditions were listed (Table 1b). The most prevalent chronic indications were cardiovascular disease (33.2%; 89/268), glaucoma (10.1%; 27/268) and diabetes (9.3%; 25/268). Collection status was significantly better in those on ART (*p* = 0.02) and treated for arthritis (*p* = 0.04) than for other indications (Table 1b). Whilst patients with glaucoma, allergic conjunctivitis and on ART were treated for those conditions alone, the majority (61.0%; 100/164) were being treated for more than one chronic condition. The top five therapeutic agents prescribed were for the cardiovascular system (14.5%; 71/491), analgesics (13.2%; 65/491), ophthalmologicals (9.4%; 46/491), antithrombotic agents (7.7%; 38/491) and nasal preparations (7.5%; 37/491). There was no statistically significant difference in the collection status between different anatomical therapeutic chemical (ATC) groups, but noncollection was higher in those collecting ophthalmological drugs (63.0%; 29/46) (Table 1c).

**TABLE 1b:** Patients’ demographics and clinical characteristics by collection status.

Disease conditions	Total (*n* = 268)		Collected (*n* = 132)		Not Collected (*n* = 136)	
	*n*	%	*n*	%	*n*	%
Cardiovascular disease	89	33.2	45	50.6	44	49.4
Glaucoma	27	10.1	11	40.7	16	59.3
Diabetes	25	9.3	15	60.0	10	40.0
Arthritis*	20	7.5	14	70.0	6	30.0
Allergic rhinitis	18	6.7	6	33.3	12	66.7
Allergic conjunctivitis	18	6.7	6	33.3	12	66.7
Gastrointestinal disease	15	5.6	7	46.7	8	53.3
HIV and AIDS*	14	5.2	11	78.6	3	21.4
Asthma	11	4.1	4	36.4	7	63.6
Epilepsy	11	4.1	5	45.5	6	54.5
Cancer	5	1.9	1	20.0	4	80.0
Other	15	5.6	7	46.7	8	53.3

* , *p* ≤ 0.05.

**TABLE 1c:** Patients’ demographics and clinical characteristics by collection status.

Anatomical therapeutic chemical	Total (*n* = 491)		Collected (*n* = 245)		Not collected (*n* = 246)	
	*n*	%	*n*	%	*n*	%
Cardiovascular drug	71	14.5	34	47.9	37	52.1
Analgesic drug	65	13.2	36	55.4	29	44.6
Ophthalmological drug	46	9.4	17	37.0	29	63.0
Antithrombotic agents	38	7.7	17	44.7	21	55.3
Nasal preparations	37	7.5	14	37.8	23	62.2
Antiepileptics	31	6.3	18	58.1	13	41.9
Antihistamines for systemic use	31	6.3	13	41.9	18	58.1
Drugs used in diabetes	23	4.7	14	60.9	9	39.1
Vitamins	22	4.5	12	54.5	10	45.5
Other	127	25.9	70	55.1	57	44.9

**FIGURE 1:**
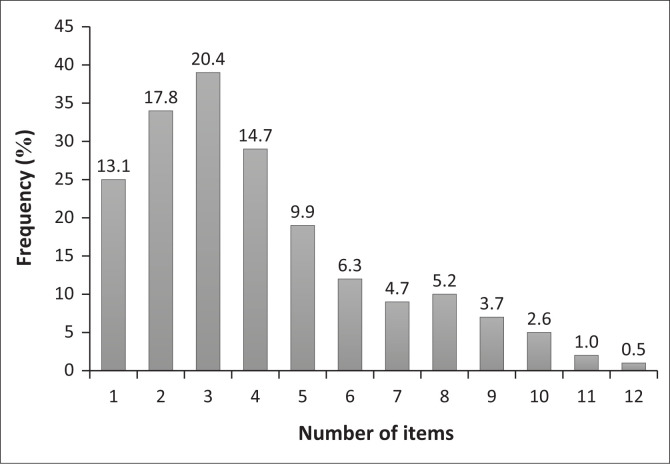
Number of items per prescription (*n* = 192).

**FIGURE 2:**
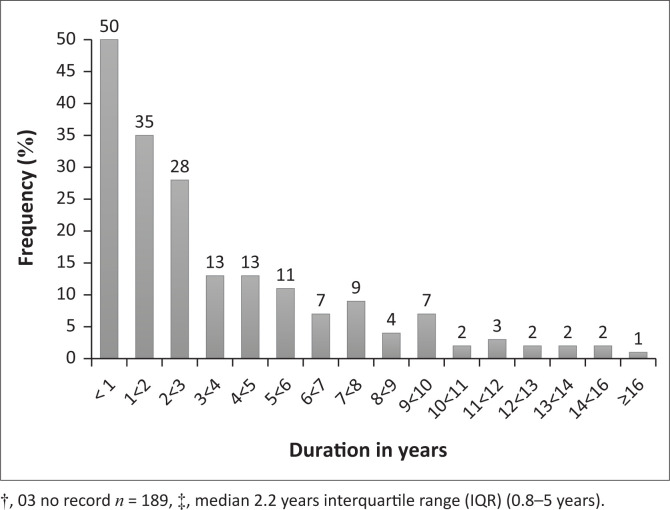
Duration on treatment (*n* = 189).†‡

**FIGURE 3:**
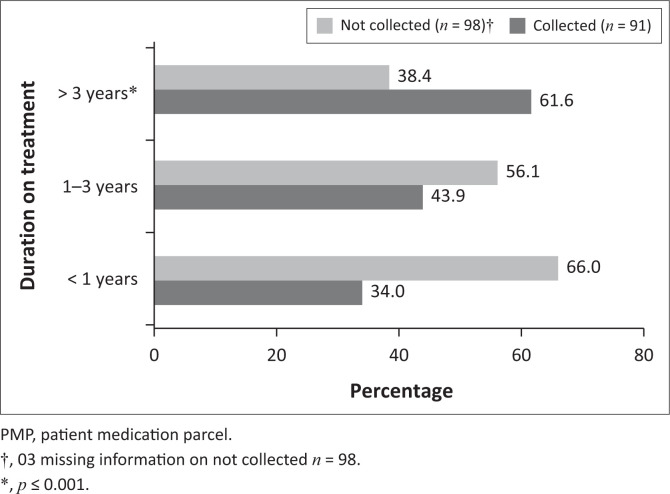
Comparisons between patient medication parcel collection and duration on treatment (*n* = 189).

The median number of items per prescription was 3.0 (IQR 2–5), with a range from 1 to 12 items. Overall, 66.1% (127/192) of prescriptions contained between one and four items (Figure 1). The proportion of collectors did not increase with the number of items per prescription (*p* = 0.65). For collectors, the median number of items per prescription was four (IQR 2–7), whilst for noncollectors it was three items per prescription (IQR 2–5) (Table 1d). Patients who had been on treatment for < 1 year, 1 ≤ 2 years and 2 ≤ 3 years accounted for 26.5% (50/189), 18.5% (35/189) and 14.8% (28/189), respectively, so the number of patients enrolled on the treatment decreased with an increase in duration of treatment (Figure 2). The median number of years on treatment, as per the prescriptions audited, was 2.2 years (IQR 0.8–5.0 years). The longer a patient stayed on treatment, the greater the chances that the PMPs would be collected, and this association was statistically significant (*p* = 0.0006). Patients who had been on treatment for longer than 3 years showed the highest proportion of collectors (61.6%, 45/73) (Figure 3). This could possibly be related to survival bias. Of 191 prescriptions for which a record of the PuP could be extracted, 107 (56.0%) were collected from hospital, whilst the remaining were either collected at contracted community pharmacies (53; 27.7%) or at a non-health facility (31; 16.2%). Patients who collected from hospital were more likely to collect (55.1%; 59/107) than those who collected from external PuPs, including community pharmacies (35.8%; 19/53) and non-health facilities (38.7%; 12/31) (*p* = 0.04) (Table 2).

**TABLE 1d:** Patients’ demographics and clinical characteristics by collection status.

Items per prescription	Total (*n* = 192)		Collected (*n* = 91)†		Not collected (*n* = 101) ‡	
	*n*	%	*n*	%	*n*	%
1	25	13.0	14	56.0	11	44.0
2	34	17.7	14	41.2	20	58.8
3	39	20.3	16	41.0	23	59.0
4	29	15.1	13	44.8	16	55.2
5–12	65	33.9	34	52.3	31	47.7

† , Collected interquartile range (IQR) (2–7) median = 4;

‡ , Not collected median IQR (2–5) = 3.

**TABLE 2:** Distribution of pick-up-points for collection of patient medication parcels.

Pick-up-point	Descriptive		Analytic				Location
	Total (*n* = 191)†		Collected (*n* = 91)		Not collected (*n* = 101)		
	*n*	%	*n*	%	*n*	%	
Hospital	107	56.0	59	55.1	48	44.9	Internal*
Private sector community pharmacies	53	27.7	19	35.8	34	64.2	External
Community-based non-health facility	31	16.2	12	38.7	19	61.3	External

**Total**	**191**	**100.0**	**90**	**47.1**	**101**	**52.9**	**-**

* , *p* ≤ 0.05.

† , 01 missing.

### Multivariate analysis

In order to adjust for possible confounding, a logistic regression model was used to identify independent factors associated with PMP collection. Two factors remained independently associated with PMP collection in the multivariable model. Patients using internal PuPs (i.e. hospital) were significantly more likely to collect their PMPs than patients using external PuPs (adjusted odds ratio [AOR] 2.1; 95% CI 1.0–4.3; *p* = 0.04), as were those below employable age, in comparison to the employed (AOR 5.6; 95% CI 1.1–28.3; *p* = 0.04). Patients who have been on treatment longer, patients with arthritis, HIV and AIDS and patients receiving multiple items were more likely to collect medication, but the association was no longer significant after adjusting for other confounders.

### Service provider database analysis

**FIGURE 4:**
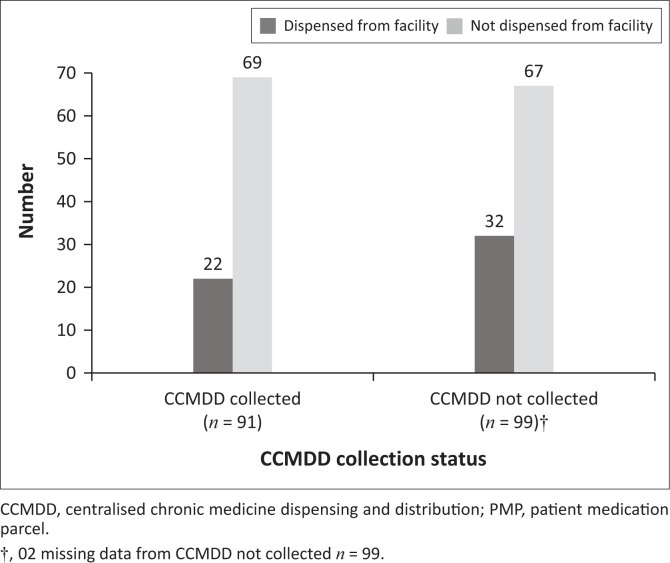
Facility versus service provider patient medication parcels collection status (*n* = 190).

The analysis of the CCMDD service provider database with patients registered under this hospital between April and September 2017 indicated that overall, 31 157 patient-months of data were recorded, of which 775 (2.5%) were classified as ‘noncollector’ patient-months. The percentage of noncollectors varied from 0.0% to 7.9% per month over the 6-month period (Table 3). Of the 91 sampled PMPs recorded as having been collected by the CCMDD service provider, 22 reflected being dispensed again from hospital stock, as shown on patients’ records, and the remaining 69 were not dispensed from hospital stock. Similarly, of the 99 sampled PMPs recorded by the CCMDD service provider as not collected, 67 were not dispensed, as shown on the hospital patient records as well, but 32 were actually dispensed to patients from hospital stock (Figure 4).

**TABLE 3:** Centralised chronic medicine dispensing and distribution patients registered April–September 2017 (*n* = 31157).

Month of 2017	Noncollectors		Collectors		Total
	Number per month	Percentage	Number per month	Percentage	Number per month
April	24	0.5	4908	99.5	4932
May	318	6.0	4954	94.0	5272
June	19	0.3	5566	99.7	5585
July	410	7.9	4797	92.1	5207
August	4	0.1	4974	99.9	4978
September	0	0.0	5183	100.0	5183

**Total**	**775**	**2.5**	**30382**	**97.5**	**31157**

## Discussion

The mean age (55 years) of the sampled patients was reflective of the current burden of chronic diseases in South Africa (Nojilana et al. 2016). By 2040, an increase in NCDs in those aged 35 years and older is predicted (Bollyky et al. 2017). The proportion of patients who collected their PMPs was highest in the youngest age group (between 0 and 14 years) and marginally declined with an increase in age. Similar to other studies done in South Africa, female patients were slightly more likely to collect than males, although the difference was not statistically significant (Hitchcock 2016; Katende-Kyenda 2018; Magadzire et al. 2017; Munyikwa 2010). By contrast, other adherence studies have found men to be more adherent than women (Lawson et al. 2020; Magadzire et al. 2017; Rolnick et al. 2013; Vetrano et al. 2017).

Those below employable age were more likely to collect their PMPs, followed by pensioners; this could be attributed to parental care. In a study conducted in the Eastern Cape, in patients collecting ART through CCMDD, there was no statistical difference in collecting PMPs between the employed and unemployed (Katende-Kyenda 2018). This study found that there was no association between patients’ employment status and adherence rate in a univariate analysis, although the unemployed were more adherent than the employed group. Work commitment was amongst the reasons identified for not collecting medicine in the WC CDU (Magadzire et al. 2017), something that the CCMDD programme aimed to achieve through increasing convenience for the employed group. However, the programme should continue to monitor adherence in all age groups, in groups with different socio-economic status and in groups being treated for different chronic conditions.

HIV was not amongst the top five listed conditions in the sampled patients collecting PMPs through CCMDD programme, despite its prevalence (Ndinda et al. 2018). The low percentage of patients on ART (12%) collecting through the WC CDU was also noted (Magadzire et al. 2015). This may be indicative of poor integration of NCDs and HIV programme management. Nonetheless, there are important lessons that can be transferred from the ART to NCD programmes, such as the need for dedicated resources and systems to facilitate access and adherence (Bradley et al. 2016; Magadzire et al. 2015). Chronic disease conditions were associated with varying adherence levels, with PMPs for HIV and arthritis most likely to be collected. In the case of diabetes, a far higher proportion of PMPs was collected (60.0%) compared with that reported by the WC CDU (33.3%) (Hitchcock 2016). Overall, the low proportion of PMPs collected for the four major causes of mortality worldwide (cardiovascular, diabetes, cancer and chronic respiratory diseases) was very concerning (Ndinda et al. 2018).

As insulin cannot be distributed by the CCMDD programme, patients with type 1 or failed type 2 cannot be enrolled. This could explain why, despite the prevalence of diabetes mellitus, antidiabetic medicines were not amongst the top five prescribed ATC classes. Analgesics were prescribed without an indication in most cases, which may reflect irrational use. This has major cost implication for health service delivery and could also contribute to possible drug–drug interactions. However, there was no statistical difference between the proportions collected per ATC level 1 classification group, although noncollection was higher for the ophthalmological drugs and nasal preparations. There have been limited studies comparing adherence to different ATC groups using level 1 classification for different disease conditions, although published studies have compared adherence within the same disease condition using ATC level 2 classifications (Choudhry et al. 2011; Lawson et al. 2020). Comparing the findings of the present study with those reported in the literature is therefore challenging.

In contrast with the WC CDU, where 56.8% of patients were being treated for only one chronic disease (Munyikwa 2010), 61.0% of the patients in the present study presented with comorbidities, with a median of 3.0 items per prescription. Patients with a comorbidity were more likely to collect PMPs, contrary to the findings in the WC CDU (Hitchcock 2016), which identified a high number of noncollectors amongst type 2 diabetes patients with comorbidities. Comorbidity has largely been associated with poor adherence to complex treatment regimens, emphasising the need to integrate chronic disease management (Vaidya, Gupte & Balkrishnan 2013; Vetrano et al. 2017). However, polypharmacy could have been masked by the poor synchronisation of prescriptions for patients presenting with comorbidities. Polypharmacy is amongst the challenges faced by people living with comorbidities (Lawson et al. 2020; Plakas et al. 2016; Vetrano et al. 2017). Patients with comorbidities have been reported to be more adherent to medication for a pre-existing condition than to treatment for a new diagnosis (An & Nichol 2013). Consolidation of CCMDD prescriptions has the potential of cost-saving from both the patient and Department of Health (DOH) perspectives, as the service provider is paid per prescription dispensed. Refill consolidation could further improve the collection of PMPs, as the patient could save on travel costs by making one trip to collect the parcels for all chronic prescriptions at the same time (Choudhry et al. 2011).

The majority of the patients had been on chronic treatment for a period less than a year, with a median of 2.2 years. The stability of these patients on chronic treatment is questionable. It has been reported elsewhere that recently diagnosed and unstable patients are being enrolled into the CCMDD programme (Magadzire et al. 2017). The present study showed that the longer a patient had stayed on treatment, the greater the chances of collection. Differential adherence has been noted between those on long-term therapy and those who have recently been initiated on therapy (Choudhry et al. 2011).

Notably, 27.4% of the sampled patients had experienced negative clinical outcomes, indicating that their chronic conditions were not yet stabilised with the treatment prescribed. Those who experienced negative clinical outcomes were less likely to collect compared to those with positive clinical outcomes. It could be that these patients may not have reached required adherence levels to be included in the CCMDD programme (Dutt 2017). The inclusion of uncontrolled patients is contrary to the CCMDD enrolment criteria, which specifically state that patients need to first be assessed as stable on chronic medication before being enrolled. Alternative interventions such as pill counts, clinical pharmacist services and coping skills training with frequent monitoring of clinical outcomes must be implemented for any patients who are identified as having an uncontrolled chronic condition (Gatwood et al. 2018; Pinto et al. 2018; Sherwood et al. 2017). Any deviation from the criteria for enrolling patients onto the CCMDD programme and consequences thereof would require further investigation.

The patients that had complicated on chronic medication whilst being managed at a lower level of care and were referred to a higher level of care should ideally be down-referred after being stabilised at the higher level of care before being enrolled into the CCMDD programme by their respective health facilities. This was identified as a gap in the patient identification process for inclusion into the CCMDD programme at higher level of care and for the decongestion of regional and tertiary facilities. Greater emphasis should be put on decongesting patients to the primary level of care, because patients residing within community area were more likely to collect compared to their counterparts residing outside community area, although the difference was statistically not significant. Furthermore, those collecting from the internal PuPs were more likely to collect compared to those collecting from external PuPs. The noncollection by those residing outside of community area may mean that these patients should have preferably been enrolled onto the programme by their respective health centres, closer to where they live. A WC CDU study also found that some patients enjoyed walking to fetch their PMPs and considered this as part of a healthy living intervention (Hitchcock 2016). Affording patients the opportunity to collect PMPs closer to where they live is expected to improve collection.

The reasons for low programme uptake by private sector community pharmacies need further investigation, as the results show that about 27.7% utilised community pharmacies. A study conducted in Europe and North America identified a lack of collaboration between providers, inadequate compensation and lack of shared vision for different levels of pharmacists’ services as some of the barriers to the utilisation of private sector community pharmacies for state services (Mossialos et al. 2015). In South Africa, those advocating for the role of the community pharmacies in the CCMDD programme are also urging for a revision of the pharmacy regulations which could support extension of such services in rural areas (Du Toit 2014). Specifically, they have called for revisions of the legislation to allow for the establishment of satellite facilities linked to community pharmacies but managed by pharmacy support personnel. The South African Pharmacy Council (SAPC) is discussing the regulatory requirement for such Pharmacy-Linked Distribution Points (PLDPs) that would not only serve as PuPs but also as wellness centres where screening tests could be provided (Du Toit 2017). The use of community-based non-health facility PuPs in this study was low, at 16.2%. Only the Methodist churches in the different community sections were identified as community-based non-health facilities for the CCMDD programme. There is an urgent need for the development of the policy on the use of community-based distribution models, because barriers and facilitators were identified in the choice of this model (Magadzire et al. 2016). Of the patients who collected from external PuPs, 35.8% and 38.7% collected from private sector community pharmacies and community-based non-health facility, respectively. This could be a true reflection of the collection status, as the external PuPs may be more adherent to the standard operating procedure (SOP) for returning uncollected parcels compared to health facility PuPs (National Department of Health 2019). This could further be attributed to the fact that external PuPs are unable to put back in stock the uncollected PMPs, as this is the state stock. There is a possibility that PMPs which are not returned by hospital-based PuPs are recorded as being collected.

Analysis done on the service provider database revealed that only 2.5% of patients did not collect their PMPs between April and September 2017, which was lower than the 8% – 12% reported in the WC CDU study (Magadzire 2016). On this basis, the overall adherence rate for this study is therefore estimated at 97.5%, which could be an overestimation, given that there is the possibility of facility PuPs not following the SOP for the handling of uncollected parcels. The medical record review further revealed that for some of the PMPs recorded as not being collected, patients were dispensed medicine from facility stock, which raises the risk of duplicate treatment. This practice has the potential for stockpiling and possible medicine wastage, indicative of weak patient information management systems (Magadzire et al. 2017). Another practice that was identified in the WC CDU study is the reusing of stock from uncollected PMPs by facilities (Magadzire et al. 2017). Health facility staff opt for this practice, as the returning of the uncollected PMPs to the service provider is regarded as an additional workload. In some instances, the patients present later than the scheduled date and it becomes easier to issue from facility stock rather than searching for the PMP. This practice has a number of implications, including the identification of patients as defaulters when in fact medicine was issued from the facility. On the other hand, there could be an overestimation of the proportion collected, as the service provider may record a parcel as being collected whereas the stock was actually placed into stock at the facility. This could also lead to poor stock accountability and financial misreporting, as the cost of medication has already been charged to the CCMDD programme. The current CCMDD process lacks visibility for PMP collection. The business model proposed by Dlamini would assist the DOH in accessing real-time collection data (Dlamini 2018).

Fruitless and wasteful expenditure related to the inappropriate handling of PMPs may not only be due to uncollected medicine, but also due to services rendered, as the DOH pays for the dispensing and logistic services. If the PMPs are not collected, but medicines are dispensed to the same patients at a health facility, the DOH is paying for the dispensing services twice (i.e. to the service provider and in the form of the salary paid to DOH-employed pharmacy personnel). In addition, if uncollected parcels are returned, the DOH is paying for the reverse logistics fees, a phenomenon that requires further in-depth investigation in order to assess the cost-effectiveness of the CCMDD programme. It is recommended that a formal study focusing on these observations be commissioned.

## Limitations

The study was limited to a single facility located in the township community and may therefore not be generalisable to other facilities in other contexts and with different patient profiles and multiple alternative PuPs linked. The study did not record per patient collection over time, as patients may miss some months during the 6-month prescription period. To calculate the duration on treatment, the current study relied on the information from patients’ clinical notes by means of medical record review. The challenge with these clinical notes is that they were not well arranged in sequence and some files were missing. The study was conducted in one facility, located in an urban district and providing regional services, making comparison of the results with facilities in rural districts and even with those providing lower levels of care challenging. The data were collected in 2017 and things may have changed since then.

## Conclusions

Similar to the findings of the studies conducted in the Western Cape and Eastern Cape provinces, where the majority of the patients collected their CCMDD PMPs dispensed from their respective health facilities (Hitchcock 2016; Katende-Kyenda 2018), this study concludes that patients collecting from internal PuPs are most likely to collect their medicines, a phenomenon that hardly achieves the decongestion of the health facilities. Mistrust of the off-site collection system, participation in the facility-based adherence clubs and support from community pharmacies facilitated patients’ choice to collect from a health facility (Hitchcock 2016). Careful consideration of patient perspectives in the implementation of the programme is required in order to maximise the potential benefits of CCMDD (Bemelmans et al. 2014).

It is further recommended that adherence be strengthened and chronic condition stability be ascertained before a patient is enrolled onto the programme. Interventions such as pill counts, clinical pharmacist services and coping skills training with frequent monitoring of clinical outcomes must be implemented for any patients who are identified as having an uncontrolled chronic condition. Down-referral to a lower level of care should be prioritised over enrolment onto the CCMDD programme. There is also an opportunity for greater use of technology that will assist with the visibility of PMPs collection at PuPs, duplicate collection by patients and identification of true defaulters for further tracking and tracing. There is also a need for more research evaluating programme implementation and uptake as well as the quality of service delivered through this innovation, as there is an opportunity for benefit from both patient and provider perspectives.

## Appendix 1: List of variables analysed, data source and type

**TABLE 1-A1:** Variables collected during data collection.

Variable	Source of data	Type
Age	Patient medical records. The Identity Document number was recorded to calculate the age as at the time of data collection.	Independent numerical continuous
Sex	Patient medical record	Independent categorical nominal
Residential address	Patient medical record	Independent and categorical
Pick-up-point	Patient medical record	Independent and categorical
Occupation	Patient medical record	Independent categorical nominal
Disease(s) being treated chronically	CCMDD prescription form (as included in the patient medical records)	Independent categorical nominal
Duration of treatment for the chronic condition	CCMDD prescription form (as included in the patient medical record) by recording prescription date within the study period that corresponded with the dispatch date against the date of the oldest prescription of the same condition as found in the patient medical records	Independent numerical continuous
Medicine prescribed categorised per therapeutic class	CCMDD prescription form (as included in the patient medical records) by listing the medicine items and classifying as per the Anatomical Therapeutic Chemical classification	Independent categorical nominal
Record of adverse events (yes/no)	Patient medical records (mainly recording treatment outcomes in terms of patient stability on treatment, e.g. uncontrolled hypertension, diabetes, asthma and episodes of seizures for epileptic patients)	Independent categorical binary
Evidence of comorbidity (yes/no)	CCMDD prescription form (as included in the patient medical records) and from information on the patient clinical charts	Independent categorical binary
Facility collection status confirmation	CCMDD prescription form (as included in the patient medical records) and from information on the patient clinical charts as endorsed by pharmacy when issuing medicine to the patient	Dependant categorical binary
Medicine listing for items per prescription	CCMDD prescription form (as included in the patient medical records)	Independent numerical continuous
CCMDD PMP collection status (collected and not collected)	CCMDD service provider data	Dependent and categorical binary outcome variable

CCMDD, centralised chronic medicine dispensing and distribution.
